# Fungal KATs/KDACs: A New Highway to Better Antifungal Drugs?

**DOI:** 10.1371/journal.ppat.1005938

**Published:** 2016-11-10

**Authors:** Karl Kuchler, Sabrina Jenull, Raju Shivarathri, Neeraj Chauhan

**Affiliations:** 1 Department of Medical Biochemistry, Medical University Vienna, Max F. Perutz Laboratories, Austria; 2 Public Health Research Institute; 3 Department of Microbiology, Biochemistry and Molecular Genetics, New Jersey Medical School, Rutgers, The State University of New Jersey, Newark; The University of North Carolina at Chapel Hill, UNITED STATES

## Introduction

According to the World Health Organization, infectious diseases stand out as the major cause of death worldwide. Although bacterial, viral, and parasitic infections appear to constitute the major threat, the clinical relevance of fungal infections has not been adequately recognized. In fact, invasive fungal infections constitute a biomedical problem of epic proportions, because a handful of human fungal pathogens claim an estimated 1.5 million lives per year [1]. Importantly, invasive fungal diseases represent leading causes of morbidity and mortality in immunocompromised individuals, particularly in patients with hematological malignancies, bone-marrow and organ transplant recipients, intensive care unit patients, preterm neonates, and patients with inborn or acquired immune deficiencies such as AIDS [2].

The vast majority of fungal infections are caused primarily by *Candida albicans*, *Aspergillus fumigatus*, and *Cryptococcus* spp. [2]. The overall mortality rate of 35%–40% for candidemia alone exceeds all gram-negative acute bacterial septicemia [3]. Importantly, pronounced inherent clinical antifungal drug resistance, especially in species like *Candida glabrata* [4], promotes a dramatic increase of infections [5, 6]. The unsolved challenge of getting fast, reliable, and accurate pathogen-specific clinical diagnosis of fungi has remained as another major impediment to successful and efficient antifungal therapy [7].

**Fig 1:**
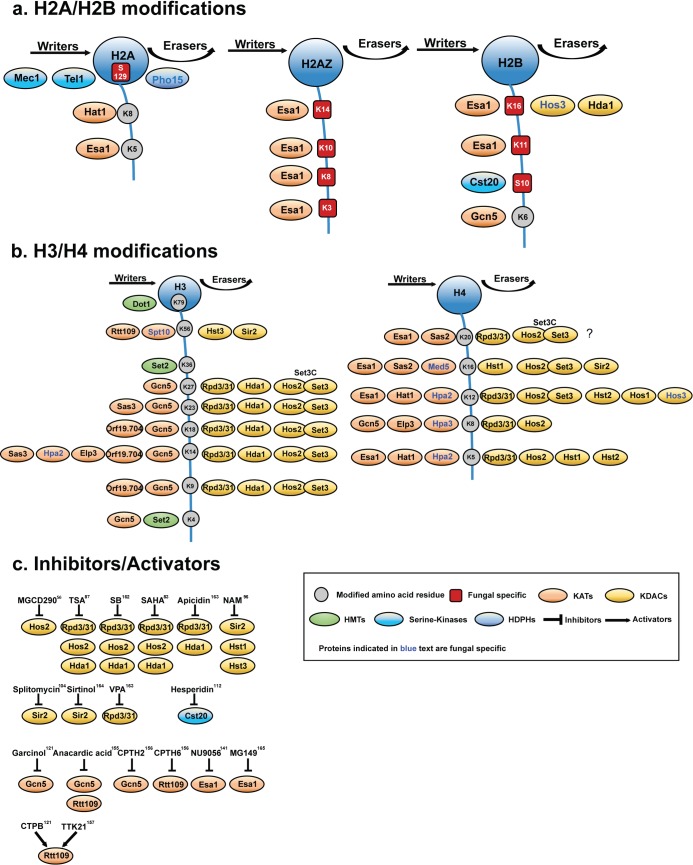
Histone modification by lysine acetylation through writers (KATs) and erasers (KDACs). Much of the mechanistic knowledge about the role of chromatin modifications in gene expression regulation comes from the nonpathogenic baker’s yeast (for excellent recent reviews, see [65–67]). Although the precise mechanisms of the interplay between writers, readers, and erasers remain ill-defined in many cases, it is fair to speculate that histone modifiers may play pivotal roles in the adaption of fungal pathogens to host immune defense. The major nucleosome building blocks, histones H2A, H2B, H3, and H4, are subject to dynamic and reversible posttranslational modifications (PTMs) by several KATs and KDACs functioning as writers and erasers of epigenetic marks. KATs like the Rtt109, which is a fungal-specific writer, and the cognate Hst3 eraser recognize the lysine residue K56 on histone H3. The KAT Esa1 acts primarily on H2A/H2B and H2AZ, with Hda1 and Hos3 acting as erasers (Panel A). By contrast, Hat1 targets mainly, though not exclusively, newly synthesized cytoplasmic histone H4 for the purpose of nuclear nucleosome remodeling during DNA damage repair [37], as well as other processes demanding nucleosome exchange. The pleiotropic KAT Gcn5 acts mainly on histone H4 and H3. Each N-terminal histone lysine can be recognized by several redundant KATs/KDACs. Histone H3 and H4 are modified by several writers and erasers in *C*. *albicans*, creating extensive combinatorial complexity and many possibilities for gene regulation depending on the cellular context. For example, the KDACs, Rpd3/31, Hda1, and the SET3C complex consisting of Set3 and Hos2 [29] act mainly on histone H3 and H4 (Panel B). Notably, kinases such as Cst20 (Panel A) and histone methyltransferases such as Dot1 and Set2 show restricted lysine specificities for histone H2B and H3, respectively. Panel C: A number of modulators of KATs/KDACs modulate (inhibit or activate) several KATs/KDACs, whereas others appear enzyme specific. Of note, no activator for KDACs have been identified for fungal KDACs, although several are known for mammalian KDACs [56,83,87,96,104,112,121,141,155–157,162–165]. TSA, trichostatin A; SB, sodium butyrate; SAHA, suberoylanilide hydroxamic acid; VPA, valporic acid; NAM, nicotinamide; CPTH2, Cyclopentylidene-[4-(4-chlorophenyl)thiazol-2-yl)hydrazine; CPTH6, 3-methylcyclopentylidene-[4-(4'-chlorophenyl)thiazol-2-yl]hydrazine; NU9056, 5-(1,2-Thiazol-5-yldisulfanyl)-1,2-thiazole; MG149, 2-(4-Heptylphenethyl)-6-hydroxybenzoic acid; CTPB, N-[4-Chloro-3-(trifluoromethyl)phenyl]-2-ethoxy-6-pentadecylbenzamide; TTK21, N-(4-Chloro-3-trifluoromethyl-phenyl)-2-n-propoxy- benzamide; HDPHs, histone dephoshorlyases; HMTs, histone methyltransferases; KATs, lysine acetyltransferases; KDACs, lysine deacetylases. Red boxes, fungal-specific modifications; grey circles, evolutionary conserved lysines in histone tails; orange ellipses, writer KATs; yellow ellipses, eraser KDACs; blue ellipses, histone dephosphorylases; cyan ellipses, histone kinases; green ellipses, histone methyltransferases.

A mere four chemical entities (polyenes, azoles, echinocandins, and flucytosine) constitute the armory of clinically relevant drugs [1]. A few variant azoles and echinocandins received recent United States Food and Drug Administration (FDA) approval, but new chemical entities are either missing or mainly experimental in nature [8]. Of note, vaccination against fungal infections is currently unavailable and heavily debated, although recent clinical trials may hold new promises as well as challenges ahead [9–11]. Interestingly enough, compelling evidence indicates that chromatin tightly controls fungal virulence and/or pathogen fitness in the host. Nucleosome remodeling and assembly pathways impact the dynamic interplay with host immune surveillance, facilitate immune evasion, as well as drive antifungal drug resistance [12]. For example, several lysine acetyltransferases (KATs) and lysine deacetylases (KDACs) control fungal virulence [13]. This suggests that KATs/KDACs modifying both histones and non-histone targets could aid in antifungal drug discovery [13, 14]. Here, we provide a comprehensive overview of chromatin modifications in human fungal pathogens, particularly those altering virulence (Table 1, Fig 1). However, owing to space constraints, we will focus our discussion on KDACs/KATs in *Candida* spp. In addition, we discuss how the modulation of KATs/KDACs in *Candida* spp. could pave the way for novel therapeutic strategies to combat fungal infections [13].

**Table 1:** Conservation of genes in human fungal pathogens encoding histone modifiers.

Catalytic subunit *Ca*	Histone target	** Inhibitors /Activators^+^	Virulence/ Fitness (mouse)	Other *Candida* spp.	Other fungal pathogens	*Sc* orthologue	Mammalian orthologue(s): modified residue	References
**KDACs**								
**Hos1/orf19.4411**	H4K12	TSA, SB, SAHA	-	*Cg*, *Ct*, *Cp*		Hos1	HDAC3/HDAC1: all four core histones	[14, 68–70]
**Hos2/orf19.5377**	specific for H3, H4 including H4K16, H4K12; in vitro: no KDAC activity?	MGCD290 (specific), TSA, SB, SAHA	attenuated (Set3)	*Cg*, *Ct*, *Cp*	*Af*, *Fo*, *Hc*, *Cn*	Hos2	HDAC3: all four core histones	[13–14, 28, 56, 69–74]
**Hos3/orf19.2772**	H4K12, H2BK16	**-**	-	*Cg*, *Ct*, *Cp*	*Af*, *Fo*, *Hc*, *Cn*	Hos3	-	[14, 69, 75–77]
**Rpd3/orf19.2834**	all four core histones, except H4K16; nonhistone: HSP90	TSA, SB, SAHA, Apicidin, VPA	-	*Cg*, *Ct*, *Cp*	*Af*, *Fo*, *Hc*, *Cn*	Rpd3	HDAC1/HDAC2: all four core histones	[31, 40, 69–70, 74, 78–84]
**Rpd31/orf19.6801**	all four core histones, except H4K16	TSA, SB, SAHA, Apicidin	attenuated	*Cg*, *Ct*, *Cp*	*Af*, *Fo*, *Hc*, *Cn*	Rpd3	HDAC1/HDAC2: all four core histones	[31, 40, 69–70, 74, 78–82, 161]
**Hda1/orf19.2606**	specific for H3, H2B including H3K9, H3K18, H2BK16; nonhistone: HSP90	TSA, SB, SAHA, Apicidin	-	*Cg*, *Ct*, *Cp*	*Af*, *Fo*, *Hc*, *Cn*	Hda1	HDAC6: all four core histones	[31, 74, 77, 84–91]
**Hst1/orf19.4761**	H3, H4 including H4K5	NAM	-	*Cg*, *Ct*, *Cp*	*Af*, *Fo*, *Hc*, *Cn*	Hst1	SIRT1/SIRT3: H4K16, H3K9	[82, 88, 92–96]
**Hst2** * **/orf19.2580**	H4K5, H4K12	NAM	-	*Cg*, *Cp*, *Ct*	*Af*, *Fo*, *Hc*, *Cn*	Hst2	SIRT3/SIRT2: H4K16, H3K9	[82, 94, 96–100]
**Hst3/orf19.1934**	H3K56	NAM	decreased	*Cg*, *Cp*, *Ct*	*Af*, *Fo*, *Hc*, *Cn*	Hst3/Hst4	SIRT3: H4K16	[25–26, 82, 96, 101]
**Sir2/orf19.1992**	H4K16, H3K56	Splitomycin (specific), NAM, Sirtinol	-	*Cg*, *Ct*, *Cp*	*Af*, *Fo*, *Hc*, *Cn* ^*a*^	Hst1 (Blast Sir2 higher identity)	SIRT1: H4K16, H3K9	[15, 69, 77, 83, 94, 100, 102–104]
**orf19.2963 (uncharacterized)**	-	-	-	*Cg*, *Ct*, *Cp*	*Hc*	Hst2	-	
**HMTs**								
**Set1/orf19.6009**	H3K4	-	decreased	*Cg*, *Ct*, *Cp*	*Af*, *Fo*, *Hc*,*Cn*	Set1	SETD1a/SETD1b:H3K4	[15, 27, 82, 105–106]
**Set2/orf19.1755**	H3K36	-	-	*Cg*, *Ct*, *Cp*	*Af*, *Fo*, *Hc*, *Cn*	Set2	SETD2: H3K36	[27, 82, 107–108]
**Dot1/orf19.7402**	H3K79	-	-	*Cg*, *Ct*, *Cp*	*Af*, *Fo*, *Hc*,*Cn*	Dot1	DOT1L: H3K79	[27, 82, 107–108]
**Serine-Kinases**								
**Cst20/orf19.4242**	H2BS10	Hesperidin (developed for Mst1)	attenuated	*Cg*, *Ct*, *Cp*	*Af*, *Hc*	Ste20	MST1: H2B14	[75, 109–112]
**Mec1/orf19.1283**	H2AS129	-	-	*Cg*, *Ct*, *Cp*	*Af*, *Hc*, *Cn*	Mec1	ATM: H2AX139	[113–114]
**Tel1/orf19.5580**	H2AS129	-	-	*Cg*, *Ct*, *Cp*	*Af*, *Fo*, *Hc*, *Cn*	Tel1	ATR: H2AX139	[113–115]
**HDPH**								
**Pho15/orf19.4444**	H2A (*in vitro*)	-	competitive fitness normal	*Cg*, *Ct*, *Cp*	*Af*, *Fo*, *Hc*, *Cn*	Pho13	-	[27, 116–117]
**KATs**								
**Gcn5/ orf19.705**	H2BK6, H3(K4, K9, K14, K18, K23, K27), H4K8	Garcinol, Anacardic acid, CPTH2	decreased	*Cg*, *Ct*, *Cd*, *Cp*	*Af*, *Fo*, *Hc*, *Cn*	Gcn5	KAT2A and KAT2B: H3K9, H3K14, H3K18,	[41, 57, 74, 118–127]
**Hat1/ orf19.779**	H2AK8, H4(K5, K12)		decreased	*Cg*, *Ct*, *Cd*, *Cp*	*Af*, *Fo*, *Hc*, *Cn*	Hat1	HAT1/KAT1: H2AK5, H4K5, H4K12	[37, 127–132]
**Elp3/ orf19.7387**	H3K14, H4K8			*Cg*, *Ct*, *Cd*, *Cp*	*Af*, *Fo*, *Hc*, *Cn*	Elp3	ELP3/KAT9: H3K14, H4K8	[127, 133–134]
**Hpa2/Hpa3/orf19.6323**	H3K14, H4(K5, K12)			*Ct*, *Cd*, *Cp*	*Af*, *Fo*, *Hc*, *Cn*	Hpa2	-	[135–136]
**Hpa3/Hpa2/orf19.6323**	H4K8			*-*		Hpa3	-	[135]
**Med5 (Nut1)/orf19.1808**	H4K16			*Cg*, *Ct*, *Cd*, *Cp*	Af, Fo, Cn	Nut1	-	[137–138]
**Esa1/ orf19.5416**	H2A(K5, K8), H2B(K11, K16), H2AZ(K3, K8, K10, K14), H4(K5, K12, K16, K20)	NU 9056, MG149		*Cg*, *Ct*, *Cd*, *Cp*	*Af*, *Fo*, *Hc*, *Cn*	Esa1	TIP60/KAT5: H3K14, H4K5, H4K8, H4K12, H4K16	[15, 17, 57, 127, 130, 139–141]
**Sas2/orf19.2087**	H4(K16, K20)			*Cg*, *Ct*, *Cd*, *Cp*	*Fo*	Sas2	KAT8: H4K16, H4K5, H4K8	[139, 142–144]
**Sas3/orf19.2540**	H3(K14, K23)			*Cg*, *Ct*, *Cd*, *Cp*	*Af*, *Hc*, *Cn*	Sas3	KAT6: H3K14	[15, 127,145–146]
**Nat4/orf19.4664**	H2A, H4			*Cg*, *Ct*, *Cd*, *Cp*	*Af*, *Fo*, *Hc*	Nat4	NAA40: H4, H2A	[147–148]
**TafII250 (Taf1)/orf19.7354**	H3, H4			*-*	*Af*, *Fo*, *Hc*, *Cn*	Taf1	KAT4	[127, 149–150]
**Rtt109/orf19.7491**	H3K56	Anacardic acid, CPTH6, C646 /CTPB^+^, TTK21^+^	decreased	*Cg*, *Ct*, *Cd*, *Cp*	*Af*, *Hc*, *Cn*	Rtt109	p300: H3K56	[25, 121, 125, 151–158]
**orf19.7074**	H3(K9, K14, K18)			*Cg*, *Ct*, *Cd*, *Cp*	*Af*	Sgf29	SGF29: H3K14	[159]
**Spt10/orf19.2361**	H3K56			*Cg*, *Ct*, *Cd*, *Cp*	*Af*, *Cn*	Spt10	-	[160]

Abbreviations: KDACs: lysine deacetylases; HMTs: histone methyltransferases; HDPH: histone dephosphorylase; KATs: lysine acetyltransferases; TSA: trichostatin A; SB: sodium butyrate; SAHA: suberoylanilide hydroxamic acid; VPA: valporic acid; NAM: nicotinamide; CPTH2: Cyclopentylidene-[4-(4-chlorophenyl)thiazol-2-yl)hydrazine; CPTH6: 3-methylcyclopentylidene-[4-(4'-chlorophenyl)thiazol-2-yl]hydrazine; NU9056: 5-(1,2-Thiazol-5-yldisulfanyl)-1,2-thiazole; MG149: 2-(4-Heptylphenethyl)-6-hydroxybenzoic acid; CTPB: N-[4-Chloro-3-(trifluoromethyl)phenyl]-2-ethoxy-6-pentadecylbenzamide; TTK21: N-(4-Chloro-3-trifluoromethyl-phenyl)-2-n-propoxy- benzamide; *Ca*: *Candida albicans*; *Cg*: *Candida glabrata*; *Ct*: *Candida tropicalis*; *Cp*: *Candida parapsilosis Af*: *Aspergillus fumigatus*; *Cn*: *Cryptococcus neoformans*; *Fo*: *Fusarium oxysporum*; *Hc*: *Histoplasma capsulatum*.

Source for orthologues in *Candida* spp.: *Candida* genome database (CGD) http://www.candidagenome.org/; Source for orthologues in other fungal pathogens: blast performed at EnsemblFungi http://fungi.ensembl.org/index.html, *Saccharomyces* genome database (SGD) http://www.yeastgenome.org/ and CGD.

a: In *Sc* Sir2 is a paralog of Hst1. All blast hits from other fungal pathogens showed higher identity to CaHst1 than to CaSir2.

+ KAT activators.

* majority of targets are cytoplasmatic [34].

** Most of the inhibitors/activators for respective mammalian KATs.

## Chromatin Modifications in Adaptive Gene Regulation and Virulence

The protein components of a eukaryotic chromosome include a wide variety of DNA-binding proteins required for fundamental cellular functions such as DNA replication, recombination, and repair, as well as adaptive gene regulation. Many proteins undergo reversible posttranslational modifications (PTM), among others, including acetylation, methylation, phosphorylation, sumoylation, or ubiquitination [15]. For instance, lysine residues in the amino tails of histones are frequently modified by either acetyl or methyl groups. These PTMs of histone tails constitute the epigenetic “histone code” recognized by reader and writer proteins that regulate gene expression [16]. Of note, histone modifications can also have nonepigenetic functions. In fact, there is accumulating evidence that histone modifications not only form a code but also modulate biological processes in a context-dependent manner through dedicated chromatin signaling pathways in physiology and pathology [17]. Indeed, proteomic approaches show that acetylation at ε-groups of lysine residues is a ubiquitous PTM in prokaryotes [18], plants[19], fungi [20], *Drosophila melanogaster* [21], and human cells [22], affecting chromatin function perhaps due to neutralization of the lysine charge [23]. The addition and removal of acetyl groups to lysine residues is catalyzed by evolutionary conserved KATs and KDACs, respectively (Fig 1). Although lysine acetylation was first reported for histones [18, 21, 24], it is now known to occur on non-histone proteins, including transcriptional regulators, and proteins involved in metabolism or stress signaling. Excitingly, the genetic and chemical manipulation of KAT/KDAC activities in *C*. *albicans* disclosed a function in fungal virulence [13, 25, 26].

The *C*. *albicans* genome harbors eight putative KATs and twelve KDACs [27], which have been evolutionary conserved in fungal species, including most major fungal pathogens such as *A*. *fumigatus* or *Cryptococcus neoformans* (Table 1). However, the progress in understanding their function in species other than *C*. *albicans* has been slow, primarily due to lack of tools or significant mechanistic data on KDACs/KATs. However, a plausible scenario indicates that fungal KATs/KDACs act in close cooperation with dedicated transcriptional regulators, thereby forming a dual-layer network of chromatin-mediated transcriptional control [27–30]. Indeed, the importance of lysine acetylation in host–pathogen interactions or fungal morphogenesis is beginning to emerge. For instance, inhibition of the KDACs Hda1 and Rpd3 in *C*. *albicans* blocks Hsp90-dependent antifungal resistance [31]. Likewise, genetic ablation of the KDAC Set3, a component of the SET3C complex, triggers hyperfilamentation of *C*. *albicans* but also strongly attenuates virulence [28]. Moreover, *C*. *albicans* cells lacking the KAT Rtt109 [25, 26] and the KDAC Hst3 [26] are highly sensitive to genotoxic agents and antifungal echinocandins [26]. Furthermore, Hst3 [32], Hda1, and Rpd3 [33] are also intimately involved in morphogenetic changes such as white-opaque switching, which is thought to impact host-niche occupancy as well as antifungal susceptibility of *C*. *albicans* [34, 35].

The evolutionary conserved KAT Hat1, a prototypical KAT, facilitates DNA damage repair of double strand breaks in mammals [36] and in *C*. *albicans* [37]. Interestingly, KATs also play important roles in the morphogenetic yeast to hyphae transition [28, 29], biofilm formation, and drug resistance [38–40], as well as virulence [38]. Likewise, genetic ablation of Gcn5, a highly conserved pleiotropic fungal KAT, strongly debilitates virulence [41]. Importantly, Hat1 recognizes a specific set of lysine residues on histones tails, the equivalent residues of which are either absent or not modified by mammalian orthologues, suggesting that fungal Hat1 inhibitors are unlikely to affect the mammalian Hat1, making it especially suitable as potential antifungal target.

## Non-histone Lysine Acetylation in Host-Pathogen Interactions

Interestingly, lysine acetylation of non-histone target proteins is increasingly recognized as a means to regulate cellular processes. Fungal acetylome data are just emerging [20], and it will be exciting to identify virulence modifiers from these genome-wide datasets. Interestingly, acetylation appears abundant in mitochondria [42]. However, it is not clear whether acetylation of mitochondrial proteins takes place in the cytosol before their mitochondrial import or inside mitochondria? How the acetylation status influences mitochondrial function and nuclear cross-talk or even two-component signaling pathways that regulate fungal virulence [43] remains open. Notably, mitochondria and intrinsic signaling pathways play key roles in fungal pathogenesis [43, 44], but a link of acetylation, mitochondria, and virulence remains to be discovered.

Notably, chromatin-related gene regulation contributes to *Candida* spp. survival in the human host [45] or even inside innate phagocytes. For example, during invasion of dendritic cells by *C*. *albicans*, both host and fungal chromatin experience complex modifications that regulate the magnitude of the inflammatory immune response but also the susceptibility of pathogens to immune defense [46]. Interestingly, prominent bacterial pathogens also exploit histone modifications to promote their intracellular replication or to evade host immune defense [47]. For example, *Shigella flexneri* induces its own uptake by modifying the host actin cytoskeleton [48]. *Borrelia burgdorferi* [49] and *Mycobacterium tuberculosis* [50] employ similar strategies to aid their persistence in human host cells.

## Using KATs/KDACs Modulators as Novel Antifungal Drugs

A limited arsenal of antifungals inhibit pathogen growth through fungistatic and/or fungicidal mechanisms [8, 51] by interfering with plasma membrane function (amphotericin B), cell wall glucan biogenesis (echinocandins), DNA synthesis (flucytosine), or ergosterol metabolism (azoles). Antifungal therapies are also limited because of toxicity, increasing drug resistance, as well as adverse drug–drug interactions. The former “gold standard” drug amphotericin B invariably causes severe toxicity in patients, limiting its use and effectiveness. Triazoles remain as preferred drugs because of their excellent toxicity profiles, moderate costs, and ease of oral administration [8]. However, the majority of triazoles are fungistatic rather than fungicidal, promoting the emergence of resistance [6]. Furthermore, some non–C. *albicans* species, most notably *C*. *glabrata*, display marked intrinsic resistance to triazoles and in some cases even cross-resistance to echinocandins [5]. Nonetheless, the fungicidal echinocandins have been outstanding drugs, but their use is also limited due to poor oral bioavailability, its ineffectiveness against *C*. *neoformans* or invasive aspergillosis [6], as well as high cost. Furthermore, recent reports indicate dramatically increasing prevalence of echinocandin-resistant *Candida* isolates [5, 52]. This is a serious matter of concern, especially because these species are increasingly recovered among bloodstream clinical isolates [5]. Remarkably, the incidence of echinocandin-resistant *C*. *glabrata* at certain medical centers in the US increased from 2%–3% in 2001 to more than 13% in 2010 [52]. Furthermore, the identification of multidrug-resistant (azoles and echinocandins) *C*. *glabrata* isolates [5] has set off the alarm bells, because treatment options for patients infected with such strains have become limited. Thus, the efficient antifungal therapy is hampered by a deadly combination of limited antifungal drug entities, increasing occurrence of bloodstream fungal infections, and emerging resistance, underscoring the critical need for discovering new types of antifungal drugs.

Of note, modulators of KATs/KDACs have received considerable attention as novel therapeutics in noninfectious disease settings, because protein acetylation is affected in several types of cancer and neurodegenerative diseases [53–55]. Hence, several KDAC inhibitors are currently in development as anticancer drugs or even in clinical use [53–55]. For example, MGCD290, a fungal KDAC inhibitor, proved active in combination with fluconazole and echinocandins against drug-resistant *Candida*, as well as filamentous fungi [56–57]. The best-known KDAC inhibitor trichostatin A (TSA) increases the susceptibility of *Candida* spp. to azole antifungals [31, 40, 58]. This synergy may arise from inhibitory effect of TSA on ergosterol biosynthesis or from the SET3C KDAC complex, because TSA is a regulator of Set3, which controls protein kinase A (PKA) signaling through Efg1 [28]. Hence, as outlined in Fig 1 and Table 1, exciting new data keep emerging. However, more efforts are needed to delineate the molecular mechanisms of drugs controlling activity of fungal KATs/KDACs.

## Conclusions and Outlook

Fungal infections are associated with astronomical annual Medicare costs, exceeding billions in Europe or the US, thus causing enormous economic burdens to already strained healthcare systems. Hence, current efforts in drug discovery are obviously lagging behind the need for improved antifungals. Unfortunately, the fundamental roles of KATs/KDACs in fungal pathophysiology, gene regulation, and/or adaptive genetic/epigenetic changes have not yet attracted enough attention in antifungal drug discovery. Moreover, among other roadblocks on the antifungal innovation highway, the academic setting has been struggling with insufficient funding from public and private bodies, thus further impairing the translation from basic science to application. For instance, grant support for fungal pathogen research falls several orders of magnitude below the levels of prominent bacterial or parasitic pathogens (http://www.gaffi.org/ and https://gfinder.policycures.org/PublicSearchTool/). Importantly, major pharma companies no longer entertain large-scale targeted antifungal discovery, partly because of high costs, limited number of validated targets, and high propensity of adverse toxicity owing to the eukaryotic nature of fungal pathogens. Importantly, the long-standing hesitation to exploit nonessential fungal genes as antifungal targets needs a careful reevaluation. Actually, a genetic argument predicts that essential genes may in fact even be poorer targets due to risks of drug resistance development, particularly in prophylactic settings or when overused. In fact, any gene affecting fungal fitness or adaptive changes in the host, irrespective of whether a fungal or a host gene could serve as a proper antifungal target [59]. Of note, all antifungal drugs target fungal growth in the host. However, there is increasing and compelling evidence that modulating the amplitude and magnitude of the host inflammatory immune response can be beneficial for the outcome of invasive fungal diseases [60–62]. Thus, chromatin-mediated adaptive changes during fungal pathogen host interplay opens new windows of opportunities and may hold great promises for future antifungal drug discovery.

Targeting fungal KATs/KDACs as a therapeutic strategy could also offer decisive advantages. First, fungal KATs/KDACs are structurally less well conserved, and some of the modifications are exclusively found in fungi, minimizing the risk of immune toxicity (Table 1, Fig 1). Second, the expansion of genome-scale genetic technologies, especially CRISPR/Cas9 approaches [63], makes it feasible to use dual-systems biology approaches to decipher the dynamic underlying host–pathogen relations [64] but also to better understand molecular mechanisms of KDAC/KAT functions under host immune surveillance. Of course, potential risks exist as well, because drug-mediated KDAC/KAT modulation may also lead to hyper-virulence phenotypes. For instance, blocking fungal KATs/KDACs can debilitate drug resistance but could otherwise lead to hypervirulence, owing to fitness gain in vivo due to inefficient recognition by immune surveillance [38]. Of note, virulence data on the role of other important chromatin or histone regulators mediating reversible phosphorylation and/or methylation of histones are unavailable for most fungal pathogens (Table 1). Thus, it is tempting to speculate that these genes will most likely expand the potential pool of suitable antifungal drug targets. Finally, another underexplored area is the role of non-chromatin, non-histome proteins modified by KDACs/KATs or other chromatin modifiers (Table 1). Interestingly, recent evidence indicates that non-histone targets of KATs may also play fundamental roles in fungal virulence and drug resistance [14], opening yet another new window of opportunity in antifungal drug discovery.
